# Re-Emerging Bacterial Pathogens, Resistance Genes and Promising Bioindicators in Raw and Treated Sewage—Addressing a Known Issue from a Different Angle and Perspective

**DOI:** 10.3390/microorganisms14092069

**Published:** 2026-09-16

**Authors:** Karol Korzekwa, Agnieszka Bisak, Tomasz Lepionka, Oliwia Obuch-Woszczatyńska, Klaudia Bylińska, Agnieszka Kauc, Katarzyna Skuza, Bartosz Zaborski, Małgorzata Krzyżowska

**Affiliations:** 1 Department of Medical and Environmental Microbiology, Military Institute of Hygiene and Epidemiology, 01-163 Warsaw, Poland; agnieszka.kauc@wihe.pl (A.K.); malgorzata.krzyzowska@wihe.pl (M.K.); 2 Laboratory of Hygiene and Environmental Sanitary Surveillance, Military Institute of Hygiene and Epidemiology, 01-163 Warsaw, Poland; 3 Municipal Water and Sewerage Company of Warsaw, 02-015 Warsaw, Poland; a.bisak@mpwik.com.pl (A.B.); b.zaborski@mpwik.com.pl (B.Z.); 4 Biological Threats Identification and Countermeasure Center, Military Institute of Hygiene and Epidemiology, 24-100 Pulawy, Poland; tomasz.lepionka@wihe.pl (T.L.); katarzyna.skuza@wihe.pl (K.S.); 5 Parasitology Laboratory, Military Institute of Hygiene and Epidemiology, 01-163 Warsaw, Poland; oliwia.obuch@wihe.pl (O.O.-W.); klaudia.bylinska@wihe.pl (K.B.); 6 Department of Preclinical Sciences, Faculty of Veterinary Medicine, Warsaw University of Life Sciences, 02-787 Warsaw, Poland

**Keywords:** drug-resistant bacteria, wastewater-based epidemiology, environmental hygiene, public health, prevention, pre-epidemic counteractions

## Abstract

Municipal wastewater catchments are significant sources of symbiotic, opportunistic, and drug-resistant bacteria that can acquire or enhance their resistance. This “re-emergence of threats” presents a serious public health issue, especially during crisis conditions (COVID-19). The aim of this study was to propose a sampling algorithm, identify strategically important sites within the wastewater catchment, expand the range of bacterial indicators, and rapidly confirm potential threats using in vitro diagnostics in both raw and treated sewage. A parallel objective was to evaluate the effectiveness of sequencing methods in pre-epidemic studies. The research was conducted as part of a long-term surveillance program at transportation hubs, healthcare facilities, residential complexes, and wastewater treatment plants in Warsaw, Poland. Total nucleic acid (TNA) was isolated from samples, amplified using qPCR kits, and selected samples underwent next-generation sequencing. In raw sewage, the most frequently detected regions included sequences complementary to the bacterial *vanB* gene, the Integron Verona-encoded metallo-β-lactamase (VIM) gene, and markers for *Mycobacterium tuberculosis*. Treated environmental effluents primarily contained DNA fragments complementary to the bacterial *vanB* sequence, along with genes related to resistance against oxacillin and imipenem, as well as the VIM gene and *Legionella* sp. 16S rRNA sequencing of the sewage samples revealed the presence of 12 promising bacterial indicators relevant to public health and environmental hygiene. The proposed algorithm aligns with the provisions of EU Directive 2024/3019 and in vitro diagnostic tests demonstrated the potential and utility of rapid screening analyses of wastewater during disruptions or crises, as well as in situations involving equipment shortages and logistical challenges in healthcare. The proposed expanded range of bioindicators could expedite decision-making regarding preventive measures and medical support for various counties, hospitals, and transportation hubs in large urban areas. The sequencing results further emphasized the need to broaden the scope of the bacterial indicators for environmental surveillance.

## 1. Introduction

The impact of wastewater on the microbiological quality of water has been extensively discussed, but it was often overlooked during the COVID-19 pandemic. Raw sewage contains a variety of harmful microorganisms and substances, including 13 types of viruses, 21 families of bacteria, 29 genera of micro-fungi, 3 genera of protists, 7 genera of ciliates, and 15 genera of parasites. Each year, over 38% of bacteria, along with toxins, bacterial proteins, enzymes, and xenobiotics, are released into a global sewage volume of 360 km^3^ [1].

In the large volume of wastewater, there is a significant amount of nucleic acids, which are the genetic components found in bacterial cells. This genetic information exists in both complete genomes and fragmented replicons suspended in the sewage’s water phase. The cycling of this genetic information is crucial, making sewage a continuous laboratory for genetic engineering. It serves as a valuable early-stage monitoring tool and provides important data for wastewater-based epidemiology (WBE) [2,3,4,5]. However, comprehensive and ready-made diagnostic (IVD) tests for qualitative and quantitative analyses of sewage from various sources—such as communication hubs, hospitals, and residential areas—are still scarce [6,7]. Most bacterial testing using IVD tests focuses on *Helicobacter* and *Mycoplasma* species. However, there are numerous indicator bacteria that reflect the epidemiological status of the population, which have been overlooked and should be included again in international and national standards and recommendations. Understanding the diversity of pathogens in wastewater offers insights into the threats posed by specific catchment and their contributions to wastewater treatment, epidemiology, and the evolution of re-emerging and emerging pathogens [8,9]. These pathogens spreading through wastewater pose serious risks to human health and environmental hygiene. It is essential to monitor these risks in line with the principles of the “One Health Initiative” continuously [10]. Collaborative efforts are essential for addressing gaps in scientific knowledge in microbiology. These efforts facilitate information exchange between agencies, integrate risk assessments, adapt surveillance and early warning systems, and improve education and training programs for both civilian specialists and uniformed service personnel. Currently, there is a lack of an integrated approach to the standardization of environmental microbiological surveillance [11,12,13].

One of the key milestones of Directive (EU) 2024/3019, which pertains to the years 2035, 2039, and 2045, is the implementation of rigorous and detailed controls over wastewater treatment standards. This initiative also focuses on monitoring the removal of microbial resistance determinants. To address these requirements, this paper proposes several solutions, including: (a) enhancing the gold standard for sampling; (b) developing an algorithm for monitoring municipal wastewater; (c) supplementing existing standards with new indicators; (d) providing training for services in both peacetime and crisis situations. It is well-known that during a crisis, established norms may lose their validity or become less applicable. In such times of disruption, adjustments made by Rapidly Deployable Outbreak Investigation Teams (RDOIT)—the anti-epidemic response units at both civilian and military levels—are typically implemented [14]. However, the issue of verifying currently used bacterial indicators of environmental sanitary, epidemiological, and hygienic status remains.

The significant contribution of fecal bacteria, such as *Campylobacterota*, *Bacillota*, *Pseudomonadota* phyla, *Epsilonproteobacteria* and *Clostridia*, *Bacteroidia*, Gamma-proteobacteria classes, including *Campylobacterales*, *Lachnospirales*, *Eubacteriales* and *Aeromonadales*, in soils, surface waters, and water treated for consumption is increasingly being highlighted. These bacteria are increasingly reported as dominant in sewage sludge processing, including during anaerobic sludge fermentation. As bacteria of the gastrointestinal microbiome, they can provide valuable information about the equivalent population excreting them into the sewage system. Expanding the range of indicators will enable tracking not only diseases of the gastrointestinal tract itself but also other human organs and human habits [15,16,17,18]. There is a disparity between the bacteria we test, the information we obtain, and the bacteria that actually dominate the sewage environment. Incorporating modern sequencing technologies into environmental research suggests that expanding the range of bacterial indicators will soon be possible.

For these purposes, we proposed to implement IVD tests and verify five hypotheses: (i) the entire capital city catchment area, regardless of its type, is a source of drug-resistant bacterial patterns, (ii) the frequency of resistance genes and pathogens in raw and treated sewage is not equal, (iii) qPCR tests and NGS sequencing strengthen the methodology of surveillance procedures and the range of bacterial indicators, (iv) R&D cooperation accelerates the assessment of hygiene and epidemiological risk of sewage, (v) multiplex qPCR in proposed IVD tests expands the possibilities of anti-epidemic protection of the population and strengthens the preventive effect in periods of any disruptions (crises) or without them.

## 2. Materials and Methods

### 2.1. Sampling and Sample Collection Points

Materials were collected from an area of 517 km^2^ in Warsaw, with a population of about 2 million (population equivalent—p.e.), generating 545,000 m^3^ of sewage daily from domestic, hospital, industrial use, stormwater, urban runoff, and agricultural sources. From spring 2023 to winter 2024 (two COVID-19 waves), nearly 400 samples underwent qualitative analyses, including *Legionella* spp. (165 samples) and *Mycobacterium* spp. (70 samples). All samples were collected on the same day to ensure reliability. Supportive biochemical parameters were published earlier [19]. Materials were collected from revision wells or were automatically composed for raw inflows in WTPs (WS 312; no. 4121541, WaterSam GmbH, 72336 Balingen, Germany) and for treated OFs by two autosamplers: Buhler 4011 (type BU4011.60.11111, HACH LANGE, Manchester, M50 3XW, UK) and the stationary WaterSam312 (no. 4121352, Balingen, Germany) (Table A1). Sewage samples were stored at 4 °C (±2) and analyzed within 24 h according to standard procedures [20,21,22]. The characteristics of proposed parameters (bioindicators) and collection points are given in Table 1 and Table A1.

### 2.2. Nucleic Acid Extraction and Amplification

Samples (40 mL) were centrifuged (10 min, 3000× *g*, RT) and fractioned into a water and pellet. In all these fractions, total nucleic acids (TNA) were captured, concentrated, extracted and purified (Wizard Enviro Total Nucleic Acid Kit procedure, Promega—A2991, Madison, WI 53711, USA). The quantity and quality of the TNA were estimated spectrophotometrically (Nabi UV/Vis Nano Spectrophotometer, NB1-A-180404; MicroDigital Co., Geumcheon-Gu, 08503 Seoul, Republic of Korea). The tests were carried out on aqueous and pellet phases in order to eliminate errors related to the loss of information from one of the phases. All qPCR was performed in duplicate according to fully described procedures, provided in the web references below Table 1. Amplification was performed using the QuantStudio5 Real-Time system (Thermo Fisher Scientific Inc., Life Technologies Holdings Pte Ltd., Singapore). The present valid product of qPCR was marked as “1” (Ct ≤ 37) or “0” (Ct ≥ 37) when absent. Inconclusive or doubtful results were repeated, then reclassified or rejected.

### 2.3. Sequencing of 16S rDNA

Samples for sequencing were selected from two pics of COVID-19 wave (Table A2). In some cases, samples collected from the same location during both COVID-19 waves were combined due to technical limitations. Each pool consisted of up to 100 ng of TNA taken from each single collection point representative for this pool in given year/COVID-19 wave (Table A2).

Microbial identification (99% accuracy) was performed by 16S rDNA sequencing using the MinION platform (R10.4.1 flow cell) and DNA-16S Barcoding Kit (SQK-16S114.24; Oxford Nanopore Technologies, ONT, Oxford, UK) [23]. TNA samples (21 < Ct < 36) were amplified during the initial 16SrDNA-PCR (~1500 bp fragments). The regions V1-V9 of the 16S rRNA gene were amplified, and the high-accuracy base caller (HAC) on MinKNOW^TM^ software (version 21.11.8, ONT) was used for data generation to reach optimal coverage. Barcoded libraries were purified by AMPure XP beads (Beckman Coulter Diagnostics, Brea, CA, USA). Libraries (800 ng) were loaded onto an R10.4.1 flow cell (FLO-MIN114) and subjected to 72 h of sequencing on a MinION (Mk1B) device (ONT, Oxford, UK). A total of 342 GB of raw data was generated and saved in FAST5 format. After length filtering (800–2000 bp), 9.9 million reads with a mean length of 1.579 kb (mean Q-score ≈ 14.5) were retained; no read subsampling was applied. All filtered reads were classified with Kraken 2 (v2.1.3) against the NCBI Targeted Loci 16S/18S/28S/ITS database (NCBI taxonomy 1 September 2024) using the EPI2ME wf-16s pipeline (epi2me-labs/wf-16s v1.4.0). Per-sample read counts are given in Table A3. Bioinformatic analyses were supported in particular by python package pysam v0.22.1, pandas v2.0.3, fastcat v0.20.0, minimap v2 2.28-r1209, samtools v1.21, taxonkit v0.18.0 and Kraken v2.1.3. The fast5 file has been deposited in the European Nucleotide Archive (ref. PRJEB95765).

### 2.4. Statistical Analysis and Interpretation of the IVD Tests

Descriptive statistics were performed to find the answer to the question of whether the parameter occurred and how often. Hierarchical clustering analysis was performed to find out whether and how similar the frequencies of a given parameter (Table 1) were in all CPs and whether and how similar the CPs were in terms of the frequencies of all negative features together. The cluster analysis used complete linkage for amalgamation and percent disagreement as the distance measure. Post hoc comparison of means was calculated for alfa level for critical ranges 0.05. One-Way ANOVA with multiple tests, Pearson’s correlation and hierarchical clustering were calculated and performed (Statistica v. 5.0, StatSoft, Inc., Tulsa, OK 74104, USA, and StatPlus LE 7.7.0, AnalystSoft Inc., Brandon, FL 33511, USA).

The study did not confirm the presence of bacteria using culture, microscopy, or phenotyping methods. Additionally, drug resistance traits were not validated in the cultures; minimum inhibitory concentrations (MICs) were not tested, nor were they confirmed through proteomic or transcriptomic methods. As a result, the detection of a drug resistance marker in wastewater was considered a probable indication that bacterial strains may possess resistance to the drug associated with the detected gene in the environment. Therefore, throughout the remainder of this manuscript, we will use the terms “resistance to a given drug” and “presence of a given strain, species, pathotype, genus, or group of bacteria” as potentially resistant or present. To avoid excessive use of terms like “probably,” “potentially,” or “presence is not excluded,” the terminology has been simplified as proposed by the manufacturer of IVD tests.

## 3. Results

### 3.1. Antimicrobial Components and Pathogens Circulating in Sewage Catchment

**Figure 1:**
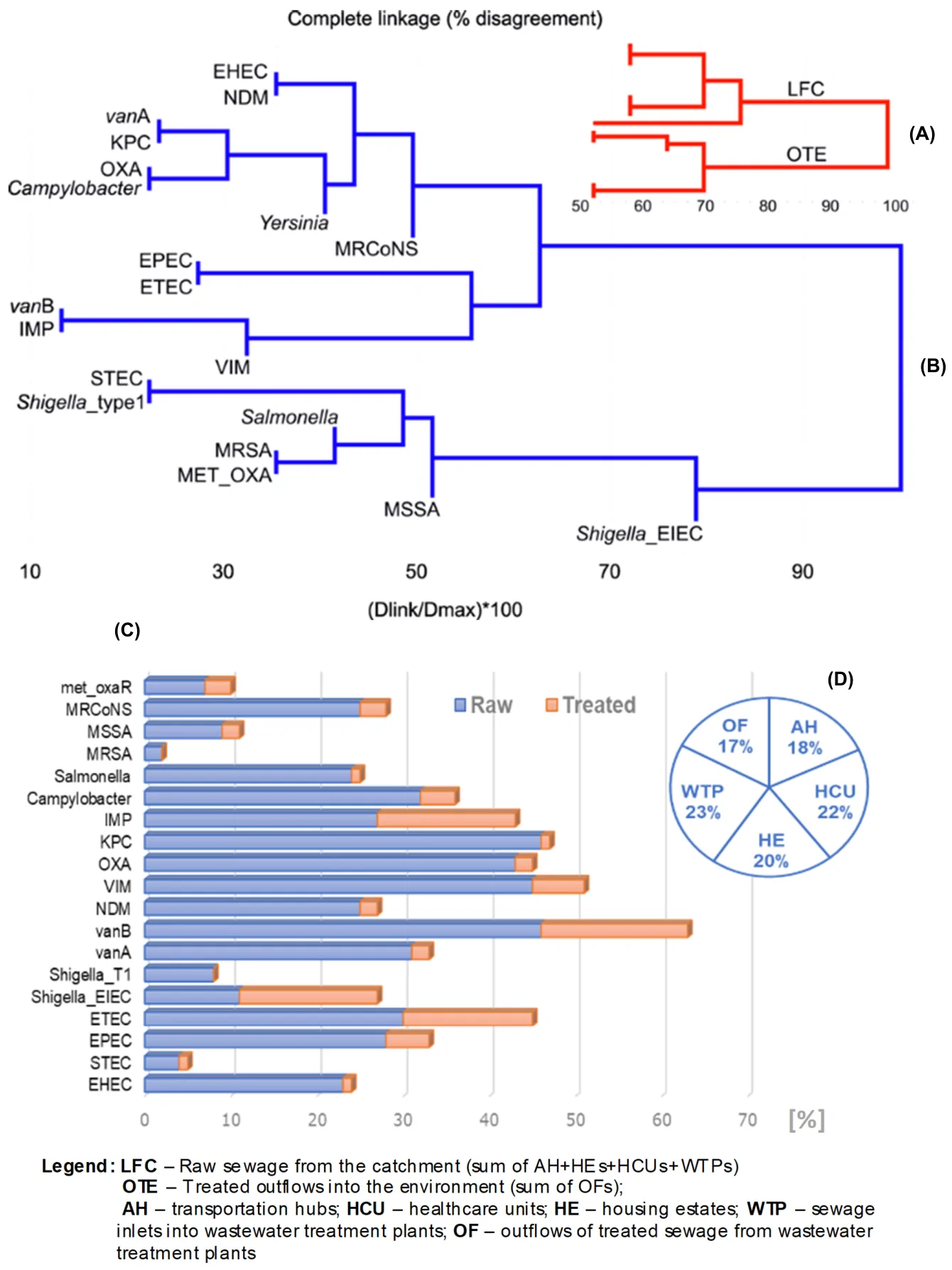
Clustering of frequencies for the presence of given gene(s) in municipal catchment (LFC) and outflows into water receivers (OTE) (**A**), individually for each trait for all collection points and whole period of investigation (**B**), with percentage of the presence of positive patterns for given trait in all CPs (**C**), and cumulative percentage of traits in given type of collection points (**D**).

The general ratio of positive results for all raw and treated samples, from the whole catchment, was 0.68 and 0.32 respectively. Taking into consideration all WTPs as 100%, the highest and cumulative load of all unfavourable traits and bacteria was represented by healthcare units (HCU, 92%), all housing estates (HE, 86%), and then transportation hubs (AH, 78%). Individual and cumulative percentage of traits in the given type of collection points is shown in Figure 1D, and detailed frequencies are presented in Table 2. Hierarchical clustering revealed two branches, individual and unique for municipal catchment load (LFC) and outflows into the environment (OTE) (Figure 1A). Based on the total environmental discharge (OTE), nearly 31% of the samples contained genetic sequences and markers associated with harmful bacteria and microorganisms that may be resistant to all β-lactams. *Salmonella enterica* was also present, though it was likely rare and found incidentally only in sample OF1.

General hierarchical analysis in all CPs revealed the most frequent co-occurrence of moderate levels of potentially vancomycin-resistant (*vanB*) and imipenem-resistance (IMP^R^) genes (Figure 1B). *Shigella*-enteroinvasive pathotypes (EIEC) were relatively the rarest presented in the samples. Simultaneously, co-occurring enterotoxigenic (ETEC) and enteropathogenic (EPEC) *E. coli* pathotypes were more common in the transportation area than elsewhere. The percentage share of drug resistance traits, pathotypes and ESKAPE bacteria in pooled CPs is presented in Figure 1C.

**Figure A1:**
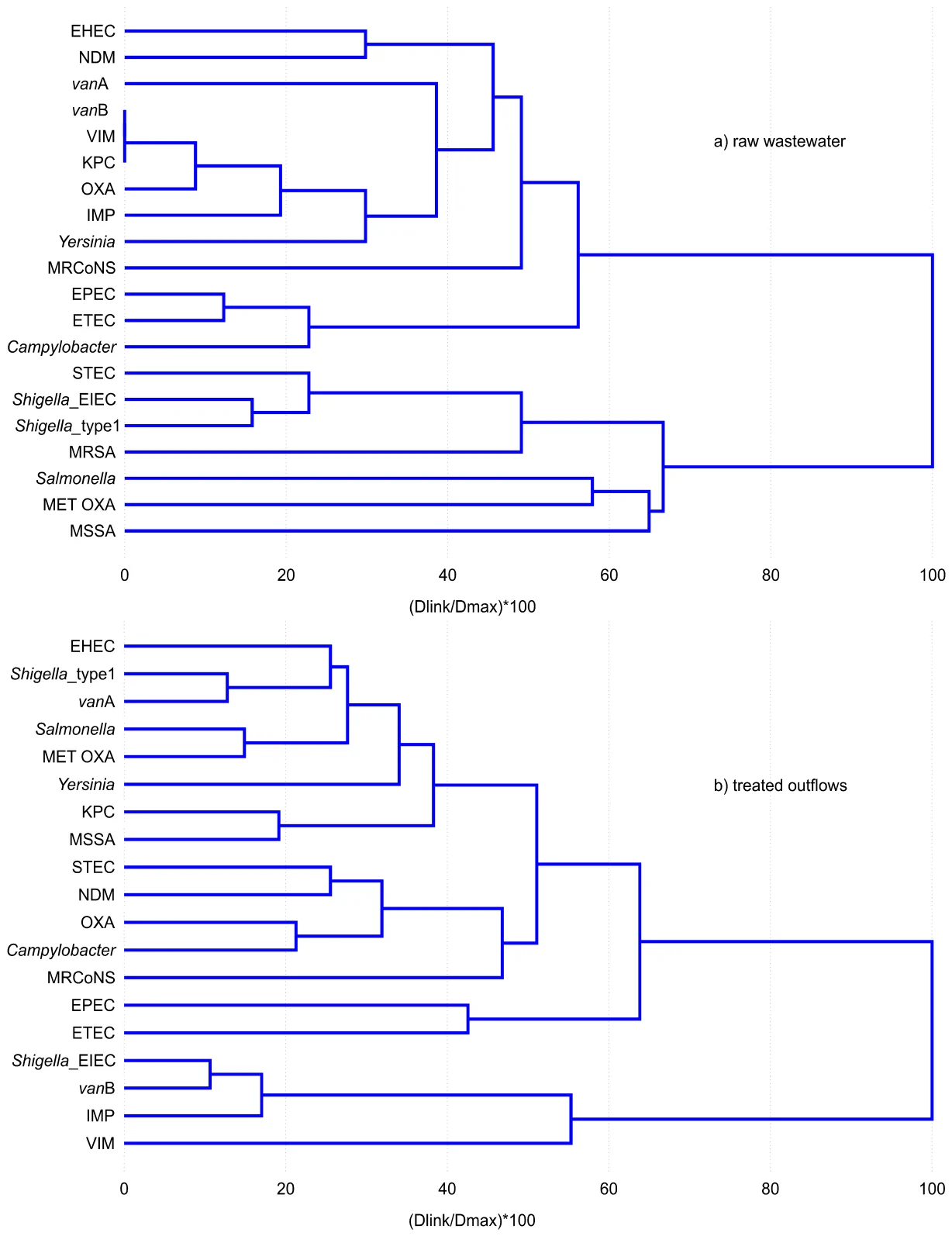
Complete linkage clustering of frequencies for the presence of investigated markers, divided separately for the whole catchment (**a**) and the whole load of discharged treated wastes (**b**) for the given, mean and representative CP (distance metric = % disagreement).

**Figure A2:**
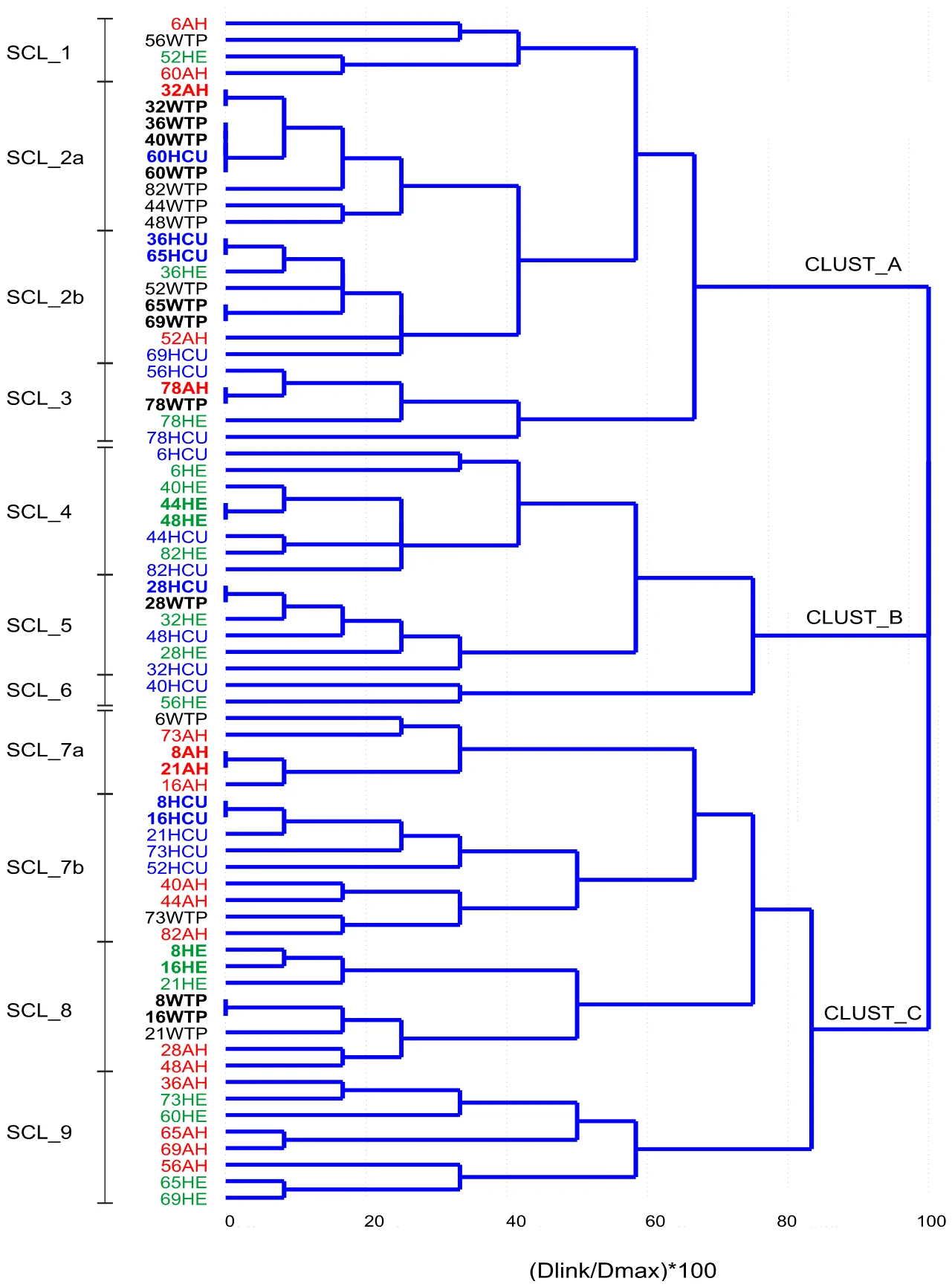
Complete linkage clustering of CPs for frequencies of given and representative investigated markers in raw wastewater (distance metric = percent of disagreement). AH—transportation hubs; HCU—healthcare units; HE—housing estates with military facilities and units; WTP—sewage inlets into wastewater treatment plants. **Bold**—the most similar CPUs in accordance with resistant traits and pathogens revealed during qPCR testing. Numbers before SCP shortcuts: 1–37—first wave of COVID-19 in 2023; numbers between 38 and 86—second wave of COVID-19 in 2024. Reports 6 to 82: respectively for 2023 (29.05., 05.06., 11.06., 22.08., 09.10., 13.11., 11.12.); 2024 (22.01., 19.02., 18.03., 22.04., 20.05., 17.06., 22.07., 19.08., 16.09., 21.10., 18.11.).

**Figure A3:**
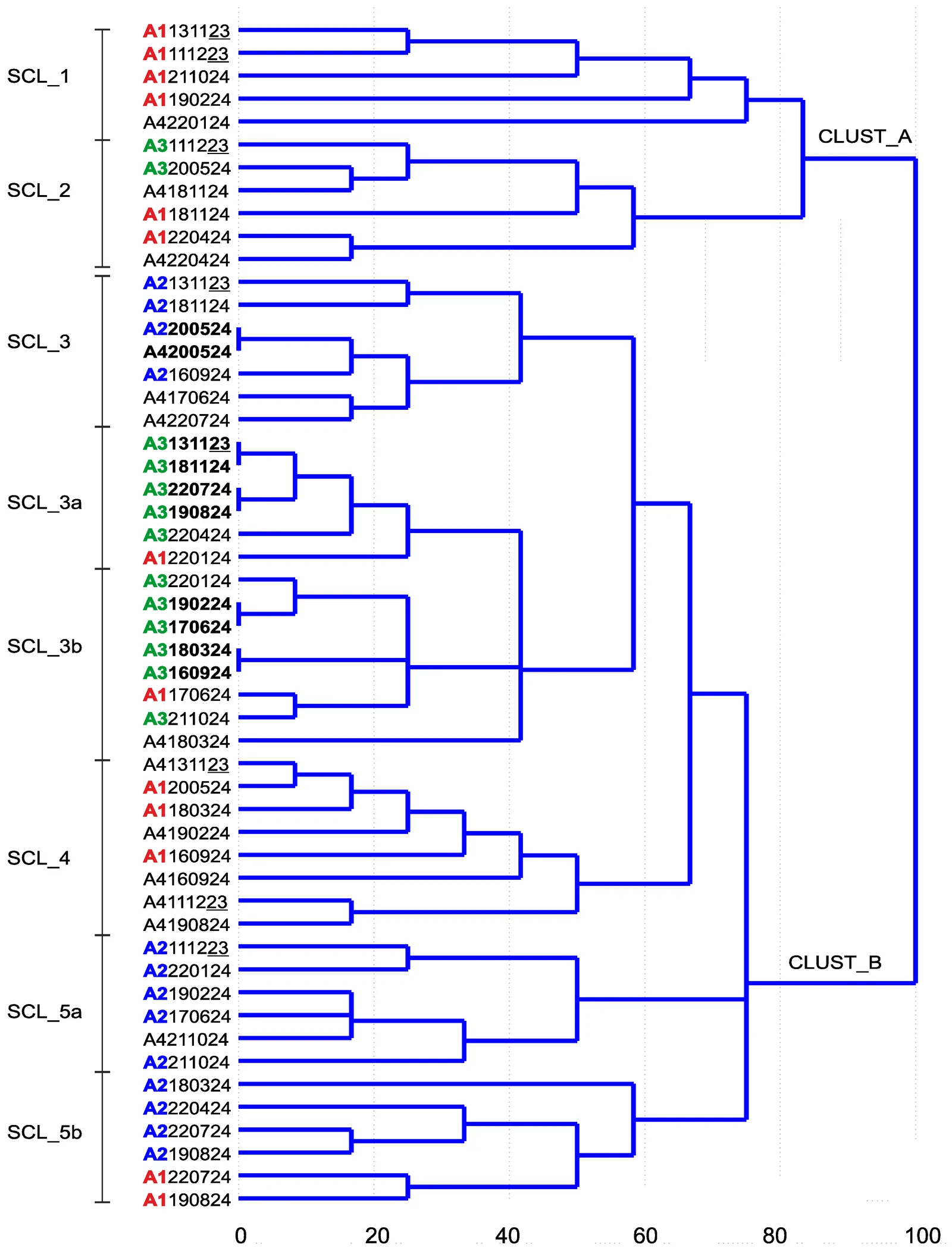
Complete linkage clustering of CPs for frequencies of given and representative investigated markers in outflows from WWTPs with dates (distance metric = percent of disagreement). **A1**—outflow from WWTP1; **A2**—outflow from WWTP2; **A3**—outflow from WWTP3; **A4**—outflow from WWTP4 (control). A2180324—mean samples recorded for outflow from WWTP2 during collection on 18 March 2023. **Bold**—the most similar CPUs in accordance with resistant traits and pathogens revealed during qPCR testing. Underlined—samples collected during first COVID-19 wave in 2023.

In raw samples, the most frequently co-occurring bacteria were moderate vancomycin-resistant enterococci (MVRE), carbapenem-resistant Verona-type strains (VIM) and carbapenemase-producing *Klebsiella pneumoniae* (KPC) (Figure A1A). For outflows, the *vanB* sequence (f = 0.94) predominantly occurred together with EIEC pathotypes (f = 0.88) (Table 2; Figure A1B). Individual hierarchical clustering of frequencies for catchments revealed heterogeneous clusters excluding SCL2a, with an advantage of WTPs, 4 for grouping HE and 7b for grouping HCU (Figure A2). Frequencies for all outflows (OFs) represented two main groups with heterogeneous Subclusters 1–3b, and cluster B represented a more homogeneous grouping, mostly samples for two WTPs, collected in 2024 (Figure A3).

### 3.2. The Presence of Legionella spp. and Mycobacterium spp. in the Catchment

**Figure 2:**
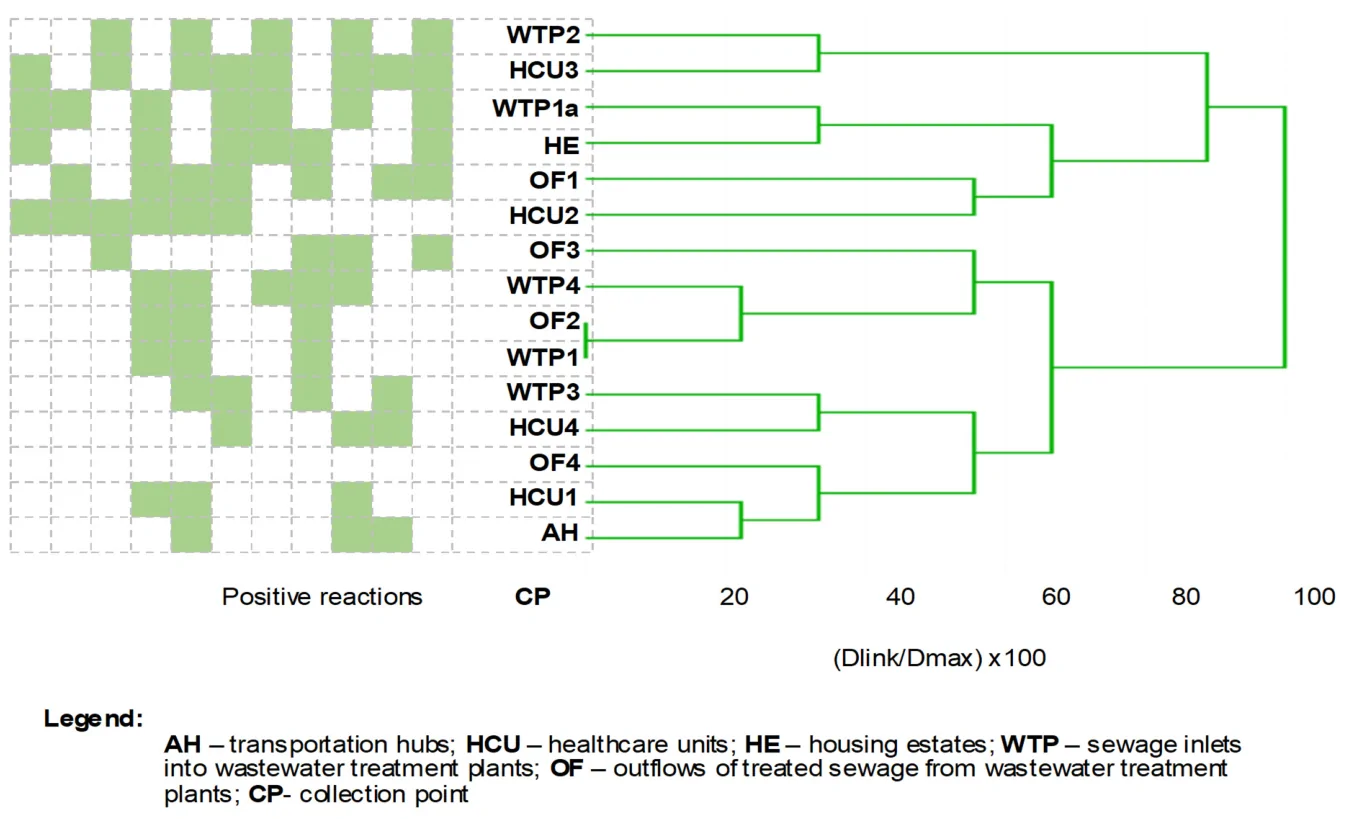
Complete linkage clustering of the frequencies for the presence of *Legionella* sp. for given and representative collection point in given and representative month during investigation (distance metric = percent of disagreement).

The presence of *Legionella* spp. was revealed for all CPs, except for the controlled OF4. This bacterium occurred most frequently in May and sporadically in February at the HCU2, WTP1a and OF1 collection points. The analysis ranked collection points based on *Legionella* spp. frequency, from 24 to 3 occurrences, respectively: HCUs > OFs > HEs > AH. The highest level of *Legionella* spp. was found at HCU3. The analysis revealed close matches between the inflow to WWTP1 and the outflow from WWTP2, as well as between WWTP4 and outflow point 3 (OF3) (Figure 2). *Legionella* spp. was isolated seven times from outflow 1 to the Vistula River.

In Warsaw’s wastewater catchment, non-tuberculous mycobacteria (NTM) were found in 71% of samples, with 19% isolated from WTP1a and nearly 10% from other inlets (Table 3). The *Mycobacterium tuberculosis* complex (MTBC) was mainly present in WTP1a, particularly *M. tuberculosis*, which accounted for 50% of MTB-tested samples. The control inlet to WWTP4 primarily contained NTM and no MTB. Frequent detection of insertion sequences suggests a possibility of isolating *Mycobacterium bovis*, *M. africanum*, *M. microti*, *M. caprae*, *M. pinnipedii*, *M. canetti*, and *M. mungi* in the wastewater entering the treatment plants.

### 3.3. Assisted Sequencing of Sewage-Derived 16S rDNA

**Figure 3:**
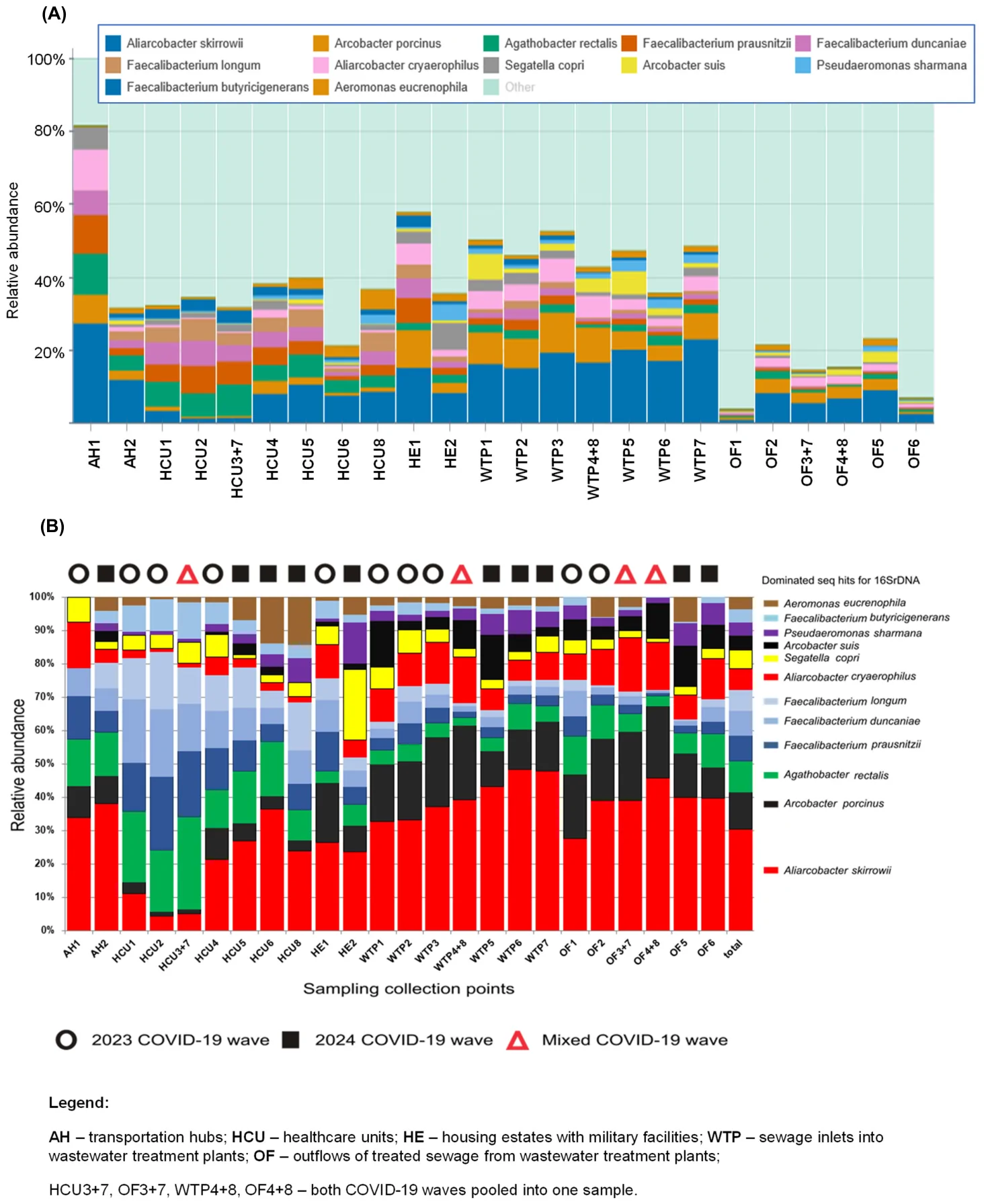
The most abundant taxa ranked by species across all samples after 16S rDNA sequencing of wastewater samples (**A**), with a special focus on the phases of COVID-19 waves during the 2023/2024 epidemics in Poland (**B**).

Pooled analysis of 24 samples allowed us to obtain the presence of 12 bacterial species (Table 4). The most abundant were *Campylobacterota*, *Bacillota* and *Pseudomonadota* phyla, *Epsilon-proteobacteria*, *Clostridia*, *Bacteroidia*, *Gamma-proteobacteria* classes, including *Campylobacterales*, *Lachnospirales*, *Eubacteriales* and *Aeromonadales* orders. Among them, the following dominated: *Arcobacteriaceae*, *Lachnospiraceae*, *Oscillospiraceae*, *Prevotellaceae* and *Aeromonadaceae* families and *Aliarcobacter* sp., *Arcobacter* sp., *Agathobacter* sp., *Faecalibacterium* sp., *Segatella* sp., *Pseudomonas* sp., and *Aeromonas* sp. (Figure 3). The ratio of relative abundance of sequenced fragments to total fragments for bacteria in samples varied from minimal in outflows (OF1, 10:90) to maximal in the transportation hub (AH1, 80:20) and mixed housing estate (HE1, 60:40) (Figure 3A). For wastewater treatment plants, the ratio was approximately 50:50. The types of species found in human-influenced urban areas (HCUs) were more like those in the transportation hub, while the species in WTPs and outflows were similar to each other.

Overall, *Aliarcobacter skirrowii* and *A. cryaerophilus* dominated in samples over *Faecalibacterium prausnitzii*, *F. duncaniae*, *F. longum* and *F. butyricigenerans*. The absolute dominator in HCUs (min. 90%) was *Faecalibacterium* sp, especially *F. prausnitzii* and *F. duncaniae* (Figure 3B; Table 4). Another local dominant was *Agathobacter rectalis*, abundant in HCUs (the first wave of COVID-19, 2023) (Figure 3B). The rare *Aeromonas eucrenophila* was in higher numbers only in HCU2 and HCU4. Another rare *Segatella copri* was present in higher numbers only in MCU. Both rare species were present in the second COVID-19 wave (2024).

Taking into consideration differences in the qualitative composition of bacteria during the 2023 or 2024 COVID-19 waves, slight differences were observed for HE (20% differences in share of *Segatella copri*), OF1 (10% differences in share of *Aeromonas eucrenophila*) and a different share of all sequenced strains for HCU1 in the 2023 than 2024 COVID-19 wave. Moreover, in healthcare units, *Aeromonas eucrenophila* was more abundant in the second wave of COVID-19 (2024).

## 4. Discussion

### 4.1. General Qualitative Analysis Using IVD Kits in WWBE

The qualitative analysis presented in this paper examines the potential presence of genetic elements (markers) and pathogens in the urban catchment area, making it the only study of its kind in Poland during the investigative period. While some researchers attempted similar analyses, their results were often fragmentary or focused on different regions [19,24,25]. The added value of the presented research is the division of the capital’s catchment area into residential (HE), healthcare units (HCU), and transportation hubs (AH), and inflows or outflows separately. The proposed algorithm for sanitary and epidemiological surveillance indicates the frequency of occurrence of the most common epidemiological threats, signaling the directions and possible sources of these threats by one laboratory technician analysing 18 samples (23 bio-factors) over 8 h using a thermocycler to process 192 wells, starting from sewage delivery. It was assumed that two 4 h shifts of RDOIT would report the results, triggering the next level of detailed analytical procedures (SOP) for the concerned entities. During crises, the results from wastewater treatment plant inputs can help assess treatment efficiency compared to standard plants operating normally. The proposed cursory methodological approach is a rapid algorithm that provides indications of the probable presence of over 60 species of resistant bacteria from 11 families in a single multidirectional analysis [26].

Proposed threats from revealed pathogens (or parameters) were evaluated, emphasizing the importance of their presence over their quantity. For example, the infectious dose of *E. coli* pathotypes, *Shigella* sp., and *Salmonella* sp. is small (1–36 cfu for some), making their presence over their number critical for epidemiological assessments [27]. For *Salmonella* spp., infectious doses range widely from 10 cfu to 10^8^ cfu [28]. Other bacteria, like *Vibrio cholerae* and *Bacillus anthracis*, require higher doses—around 10^4^ cfu—while *B. anthracis* can be infectious at one cell in animals, with human dosage estimates between 150 and 600 cells [29,30]. An even less infectious dose of *Vibrio cholerae* varies with gastrointestinal pH, between 10 and 100 cells in extreme cases [31]. *Klebsiella pneumoniae* has a low LD50 of about 10^3^ cfu, whereas *Campylobacter jejuni* needs only about 10^2^ to cause illness, and *Yersinia enterocolitica* requires up to 10^8^ cfu [32]. However, the median number of pathogens in 1 mL of a sewage sample usually oscillates between 10^2^ cfu (*Clostridium perfringens*), 10^6^ cfu (fecal enterococci), 10^7^ cfu (*E. coli*, coliform bacteria), and 10^9^ cfu (mesophiles) [33]. This indicates that the impact of diverse infectious sources is crucial for the intensity and dynamics of outbreaks (e.g., *E. coli* O1567:H7) [32]. Therefore, regardless of the usual abundance of pathogens in wastewater, simple qualitative analysis, including a multiplex qPCR method, becomes a convenient WWBE tool in tracking the extent of endemic and epidemic diseases. The presented algorithm was optimized by a library of experiences for qPCR kits during COVID-19, similar to that presented by Stobnicka-Kupiec et al. [25,33,34]. The explanation for this type of interpretation is that the occurrence of bacteria associated with the MVRE and KPC phenotype always occurred as 100% (1.0), and the MRSA phenotype is not presented as 0% (0.0). Most authors propose quantitative research with a different methodology, using the total number of all tested bacteria as the reference point instead of 100% tested molecular phenotypes. The frequency of occurrence of bacterial groups in raw sewage from Warsaw presented in the paper is twice as high as reported by other authors, especially for *E. coli* (63%) compared to 37%, and ten times higher for *Salmonella* sp., *Campylobacter* sp. and *Yersinia* sp.—39%, 90% and 77%, respectively [33].

The Warsaw catchment was a significant source of moderate-to-high levels of resistance to vancomycin (HVRE), particularly in hospitals, as a key source of *vanA* and *vanB* genes. What is more, both genes (*vanA* and *vanB*) were present in every single sample taken from WWTP influents. Furthermore, the closer the samples were taken to the final section of the Warsaw sewer network, the more frequently moderate VRE strains were observed. Iversen et al. [35] indicated that the widespread use of avoparcin in Europe is a pivotal environmental selection pressure for the high prevalence of VRE in previously susceptible enterococci isolated from sewage. In samples of raw sewage from Stockholm and Uppsala catchments, hospitals, treated sewage, and ground waters, they isolated multiresistant VRE in a quantitative ratio of 6:4:2:1. Nearly 86% of samples revealed the presence of *vanA* sequences and 69% revealed the presence of *Enterococcus faecium* [35]. Our positive qPCR results for *vanA*, *vanB* and simultaneous VIM/OXA/IMP allow us to conclude that *Enterococcus cloacae* and *Enterococcus faecium* were present in the tested samples. Bacteria that produce extended-spectrum beta-lactamases (ESBLs) were widespread in samples entering the Warsaw wastewater treatment plants (WWTPs). Frequent detections of carbapenem resistance determinants were early signs that ESBLs were spreading in the surrounding area, possibly hindering the best ESBL-related treatment for infections. Within three hours of receiving a report of wastewater containing these alarming strains, appropriate procedures were prompted on the wards. Subsequent tests at the hospitals revealed the presence of several bacteria, including *Klebsiella pneumoniae*, *E. coli*, *Pseudomonas aeruginosa*, *Acinetobacter* spp., and *Stenotrophomonas maltophilia*. The occurrence of carbapenem-resistant alarming species in the catchment area justified the development of an anti-epidemic algorithm for testing *Klebsiella pneumoniae* (ST11-15, 147) and *E. coli* (ST167, 410, 617) [26]. Using the IVD test helped quickly determine NDM strains in a transportation hub in Warsaw. This approach allowed associated researchers to focus studies on *E. coli*, *K. pneumoniae*, and *E. cloacae* originating from Egypt, India, Pakistan, Serbia, and other UAE countries [26]. For example, in Poland, the most common NDM type carries the IncFII replicon, which acts as a plasmid to spread resistance traits against beta-lactam antibiotics among different bacteria [36]. Furthermore, the frequent presence of enteroinvasive strains of *E. coli* and *Shigella* spp. in the catchment of Warsaw poses a significant risk of bacillary dysentery, severe diarrhea, fever, and potential damage to the intestinal walls [37]. In contrast, STEC *E. coli* and *Shigella* type 1 occur infrequently but show a more frequent co-occurrence at CPs. This pattern is methodologically justified, as the genetic markers *stx* (1,2) and *ipaH* form the basis of the qPCR tests conducted. The common occurrence of these bacteria at the research sites is also influenced by the close relationship between *Escherichia* spp. and *Shigella* spp., particularly *Escherichia fergusonii*, *E. coli*, and *Shigella flexneri*. Additionally, there is notable pan-genomic similarity between *E. albertii*, *Shigella boydii*, *S. sonnei*, *E. marmotae*, and *S. dysenteriae*, often forming a monophyletic clade [38].

We found that the frequent occurrence of *Staphylococcus aureus* (both MRSA and MSSA) in the same CP is rarely correlated with co-occurrence with methicillin-resistant coagulase-negative *Staphylococcus* (MRCoNS). MRSA, MSSA, and MET-OXA strains frequently grouped into related clusters, while MRCoNS clustered with *E. coli* pathotypes, *Campylobacter* spp., and *Yersinia* spp. Some authors state that MRCoNS are considered a repository of the *mecA* gene for methicillin-resistant *Staphylococcus aureus* (MRSA), and methicillin-susceptible *Staphylococcus aureus* (MSSA) can develop into MRSA [39]. That is why the significant distance of MRCoNS from MRSA and MET-OXA strains was surprising. All qPCR reactions for these patterns use probes that detect the presence of mec genes. The explanation may be the evolution of SCC elements occurring at the orfX region in *Staphylococcus* spp. This mechanism is described in detail by Miragaia [40]. The clinical tests for MRSA, MRCoNS, and MET-OXA in wastewater raise questions about their usefulness for wastewater-based epidemiology (WBE). While MSSA tests are helpful, monitoring other methicillin- and oxacillin-resistant staphylococci is challenging. We need further testing and improvements, as our understanding of MRCoNS in large cities is still limited.

### 4.2. Cycle of Mycobacterium sp. and Legionella sp. in Warsaw’s Sewage Catchment and Outflows

Despite the lack of a precise tool to assess the exact source of individual mycobacterial groups, the important information is the presence of mycobacteria, the MTB complex and the tubercle bacillus itself. MTB was not detected only in the control inflows to WWTP4 throughout the entire study period. In the control region served by this WWTP, there were no patients infected with tuberculosis, being treated, or any other decontamination and hygiene activities in hospital anti-tuberculosis wards. Unfortunately, there is no more detailed and complex information on tuberculosis for this region. The tubercle bacilli successfully migrated through the capital’s sewage system up to 20 km from the Specialist Hospital for Tuberculosis and Lung Diseases (WTP1 and WTP1a results). However, we do not know the genotypic patterns of the strains, nor can we explain why mycobacteria were more common in autumn and less common in spring. On the other hand, the high number of NTM in the catchment was not surprising due to common colonization of a wide range of ecological niches. Honda et al. [41] list the following sources of common occurrence of NTM: peat-rich soils, natural waters, drinking water, water heaters, refrigerator taps, catheters, environmental amoeba, biofilms from ice machines, heated nebulizers, and heater–cooler units, as well as seat dust from theaters, vacuum cleaners, and cobwebs. Therefore, they may appear in sewage as a consequence of inadequate sanitary and hygienic procedures in many public facilities, and runoff of environmental bacilli from the catchment area.

Diagnostic tests for *Legionella* spp. in wastewater were selected for legislative recommendations regarding the secondary circulation of treated wastewater and technological water, in accordance with the circular economy model. The frequent occurrence of *Legionella* ssp. in hospital outflows, especially during the summer months, is not surprising due to the thermophilic nature of this bacterium, the place of treatment, and the use of warm water for various purposes. Similarly, the frequent occurrence of *Legionella* sp., including *L*. *pneumophila*, in sewage treatment plant effluents, especially in August, can be explained by the thermophilic properties of this bacterium and the specificity of sewage treatment technology. The absence of these rods in the outflows from the control sewage treatment plant may be sporadic and minimal in the region consisting mainly of single-family housing estates. Moreover, the presence of *Legionella* spp. in wastewater of Warsaw’s catchment at such a relatively high percentage rate should not be surprising, given the similar living and functioning conditions of bacteria involved in wastewater treatment. *Legionella* spp. do not compete for niche space or nutritional resources with indigenous bacteria, especially in the biological treatment process, and therefore pass through the process at a reduced rate [42]. However, the degree of reduction in the number of *Legionella* spp., including *L. pneumophila*, should be confirmed.

### 4.3. Promising New Markers for Population Well-Being Surveillance and Public Health

Supportive sequencing of the 16S rDNA has proven to be a valuable tool for wastewater-based epidemiology (WBE). This technique is preferred for characterizing microbial communities because it sequences the entire gene (approximately 1500 base pairs) rather than just small fragments, which results in significantly higher taxonomic resolution. The key advantages of full-length 16S rDNA sequencing over short-read amplicon sequencing include species-level resolution, elimination of regional bias, improved accuracy in phylogenetic trees, and compatibility with long-read technologies. For these reasons, Oxford Nanopore was chosen as the most suitable platform for studying sewage microbiomes in municipal catchments.

Similarly, as we predicted, Wu et al. [43] found *Aliarcobacter* sp. and *Faecalibacterium* sp. to be abundant, and *Agathobacter* sp. one of the top 10 most abundant genera in wastewater samples. Although *Aliarcobacter* sp. is considered by this author to be a more environmental microorganism than autochthonous human gut bacteria in sewage. *Aliarcobacter skirrowii* (syn. *Arcobacter skirrowii*) may occasionally represent an aetiological factor of acute watery diarrhea and abdominal pain lasting several weeks. Predominantly, these bacteria are isolated from domestic animal intestines, raw meat, chicken, pork and beef.

*Faecalibacterium prausnitzii* is abundant in healthy people as well as in people with digestive tract disorders [44]. According to Wu et al. [43], *Faecalibacterium* sp., susceptible to alcohol, may be used as a counter-marker for population abuse of alcohol. In turn, Pennycook and Scanlan [5] pointed to a higher number of *Faecalibacterium* sp. in human gut microbiota during antituberculosis treatment with HRZE. Lopez-Siles et al. [45] pointed to *F. prausnitzii* as a biomarker to assist in gut disease diagnostics and prognostics. It may be useful to assist in the discrimination of ulcerative colitis and Crohn’s disease [41]. In some cases, the quantitative assessment of *F. prausnitzii* together with *E. coli* (F-E index) in the sewer might be a good biomarker of the number of colorectal cancer (CRC), ulcerative colitis (UC), irritable bowel syndrome (IBS) and Crohn’s disease (CD) patients in a municipal catchment, especially for monitoring of ulcerative proctitis and left-sided UC from pancolitis. Moreover, an increase in the number of *F. prausnitzii* could be associated with a high level of cortisol or α-2b interferon in sewage from healthcare units. The presence of four species of *Faecalibacterium* in municipal wastewater should undergo further consideration as a universal marker of public health, especially gastrointestinal disorders.

*Agathobacter rectalis* (syn. *Eubacterium rectale*) is a promising marker for Alzheimer’s disease (AD) symptoms and several gut microbiota dysbiosis, IBD-EIM, ASV and UC-EIM markers as well [44]. Moreover, *Agathobacter* sp. is suggested as a potential marker in wastewater for population abuse of alcohol [43]. This bacterium is equipped and armoured with a large number of genes involved in the acquisition and metabolism of polysaccharides, including environmental sensors and regulators.

*Segatella copri* (formerly *Prevotella copri*) dominates in non-Westernized populations around the world. The gut microbiome of the human population living in non-industrialized areas consists of 90% *S. copri* in contrast to 20–30% in industrialized people. Fiber-rich diets substantially contribute to the abundance of *S. copri* in populations. The presence of this bacterium is associated with a special type of rheumatoid arthritis in Westernized populations [46]. Ng et al. [47] revealed a higher abundance of *P. copri* in human GIT after SARS-CoV-2 vaccination. Moreover, Hartikainen et al. [48] pointed to the contribution of a higher number of *S. copri* in patients with irritable bowel syndrome (IBS).

*Pseudoaeromonas sharmana* (formerly *Aeromonas sharmana*) is supposed to promote plant growth caused by the presence of *nif* genes (Mo-nitrogenase). The affinity of *P. sharmana* for wastewater may be related to its isolation origin: warm river water and sediments in the area between the equator and tropical geographical latitudes. Data about their impact on human GIT is scarce or fragmented.

*Aeromonas eucrenophila*, a member of the *Aeromonas caviae* complex, does not pose a serious threat to human GIT but may be dangerous to primates. However, it may play a supportive role in the *Aeromonas hydrophila*-*Escherichia coli* system of hygienic assessment of the wastewater and impact of liquid pollutions on fresh surface water (based on our own experiences).

### 4.4. Verified Hypothesis and Implications

In line with our study, we confirmed that all capital sub-catchments, regardless of their type, are the source of drug-resistant bacteria or genetic determinants (H1 = true). The presence of drug resistance traits and pathogens differed between collection points, with a range between 17% and 23% (H2 = true). Frequencies differed between inflows and outflows (*p* < 0.001) but did not differ within inflow catchment pools (*p* > 0.05). The presence of *Legionella* sp. in treated outflows from WWTPs suggests further treatment in closed circular water systems. Additional NGS revealed and confirmed the presence of at least six valuable microbial indicators for drug-resistant surveillance, as well as GIT disorders and public health problems (H3 = true). Moreover, effective and reliable collaboration between the research institute and local authorities, in close coordination with the water and sewage company, is essential. This approach has proven crucial for the rapid development of CPs, wastewater retention time monitoring, enhancing cooperation with HCUs, and implementing various engineering barrier exclusions, which allowed us to save time and financial resources. Our model in the present research assumed the work of two people in the research (R) sector and two in development (D) in an eight-hour working mode, which we achieved (H4 = true). In the above scope of verified hypotheses, multiplex qPCR developed in IVD tests expands the possibilities of anti-epidemic protection of the population and strengthens the fast preventive effect in periods of any disruptions (crises) or without them (H5 = true).

The task was initiated to comprehensively analyze the usefulness of IVD tests for preventive civil duties, in training for civil and military teams (RDOIT), for investigative and epidemiological purposes, as well as combat use during a crisis. That is why we recommend monitoring specific pathogens in municipal wastewater, similar to the SARS-CoV-2 surveillance during COVID-19. Key actions include: (i) testing raw wastewater for EPEC, EHEC, and STEC, (ii) monitoring treated discharges from WWTPs for EIEC and ETEC, (iii) checking raw wastewater for methicillin-resistant staphylococci, vancomycin and carbapenem resistance genes, and mycobacteria sequences, (iv) testing treated effluents for *vanB* genes, (v) analyzing for *Salmonella*, *Shigella*, *Campylobacter*, and *Yersinia* in raw wastewater. Additionally, *Legionella* spp. should be included in wastewater discharge standards. Rapid PCR diagnostic methods need to be validated before use by sanitary and anti-epidemic services and military units during crises.

Moreover, we suggest further and thorough analysis of *Aliarcobacter* sp., *Faecalibacterium* sp., *Agathobacter* sp. and *Aeromonas hydrophila* as supportive public health profiling indicators. We also propose supplementing the catalogue of indicators of the well-being of the urban population with the bacteria *Aliarcobacter* sp. and *Faecalibacterium* sp., together with *Escherichia coli*, *Aeromonas* ssp., and reintroducing fecal streptococci. However, the proposed promising biomarkers and their interpretations regarding disease associations are hypothesized in this manuscript, highlighting their potential utility in public health. It is important to note that the dataset presented is not intended to be definitive, nor does it assume any relationships with the diseases discussed. To draw reliable conclusions, we need comprehensive population metadata, independent validation cohorts, absolute quantification, source tracking, and adjustments for factors such as diet, season, hydrology, and treatment processes. Therefore, these taxa are considered candidates for further exploratory studies.

## 5. Conclusions

The performed IVD tests: (a) are suitable for WWBE purposes in any disruptions or crisis conditions, addressing equipment shortcomings, media, and healthcare service deficiencies; (b) enable obtaining satisfactory results within two 4 h shifts of the mobile laboratory staff or under a regular laboratory regime twice a week; (c) serve as a support and develop the provisions of Directive EU2024/3019 for both peacetime and crisis situations; (d) facilitate faster decision-making regarding anti-epidemic measures, preventive actions, and medical support for specific districts, hospitals, and transportation hubs in large urban areas. Therefore, our results support standards that address situations beyond just those that are geopolitically stable, promoting mechanisms that remain effective outside the comfort zone in peacetime, even in the face of potential disruptions to critical infrastructure.

In the first half of this paper, we discussed the known issue of bacteria as useful bioindicators, examining it from a different perspective: the quality analysis of wastewater using IVD testing. In the second half, we introduced a methodical approach to utilizing Next-Generation Sequencing (NGS) from a pre-epidemiological standpoint. These two approaches raise important questions: Is the current selection of bacterial species adequate for preventive environmental monitoring, considering the number of species tested? Are we truly examining the genera and species that are most prevalent in wastewater? Should we consider a more extensive application of sequencing to identify new bacteria that may provide valuable insights for epidemiologists? Thus, the ‘em dash’ in the title presents a fresh perspective on a familiar topic and suggests avenues for future inquiry.

## Figures and Tables

**Table 1:** Kits used for identification and differentiation of molecular traits and pathogens in sewage samples **.

Parameter (Bioindicators)	Description of Biological Traits as the Presence of the Given Parameter	Test Ref. Number
EHEC	[*stx*1, *stx*2 and *eae*] (+)—enterohemorrhagic *E. coli* (90% of O157:H7);	7041059 ^(1)^ 7041064 ^(2)^
STEC	[*stx*1 or *stx*2] (+)—Shiga-like toxin producing *E. coli*	
EPEC	*eae* (+)—enteropathogenic *E. coli*	
*Shigella*_EIEC	multicopy *ipa*H (+)—enteroinvasive *E. coli* closely related to *Shigella* spp.	
ETEC	[*elt* and *est*1a/*est*1b] (+)—heat-labile enterotoxins for *E. coli* (O114) *	
*Shigella*_type1	[*stx*1, *stx*2, *ipa*H] (+)—*Shigella dysenteriae*, type 1	
*van* *A*	*van*A (+)—high level of vancomycin-resistant enterococci (HVRE)	7041043 ^(3)^ VS-VAN112L ^(4)^
*van* *B*	*vanB* (+)—moderate level of vancomycin-resistant enterococci (MVRE)	
NDM	*bla*_NDM_ (+)—New Delhi metallo-β-lactamase resistance type—carbapenem-resistant *Enterobacteriaceae* (CRE)	7041057 ^(5)^ VS-CPE112L ^(6)^
VIM	*bla*_VIM_ (+)—Verona integron-encoded metallo-β-lactamase resistance type carbapenem-resistant *Enterobacteriaceae* (*Klebsiella pneumoniae* and *Enterococcus cloacae*) *	
OXA	*bla*_OXA-48 like_ (+)—class D, oxacillin-resistant—carbapenem-resistant *Enterobacteriaceae* with presence of OXA-48 enzymes	
IMP	*bla*_IMP_ (+)—metallo-β-lactamase active on imipenem for cluster IMP-1 (12 subclusters) and IMP-8 (7 subclusters); carbapenem-resistant *Enterobacteriaceae*	
KPC	*bla*_KPC_ (+)—class A, serine β-lactamase activity for *Klebsiella pneumoniae* *	
MRSA	SA442/CoA (+), mobile SCC*mec*-*orfX* junction (+) and *mecA*/*mecC* (+)—methicillin-resistant *Staphylococcus aureus*	7041066 ^(7)^ VS-MSA112L ^(8)^
MSSA	SA442/CoA (+), SCC*mec*-*orfX* (+)—methicillin-sensitive *Staphylococcus aureus*	
MRCoNS	*mecA*/*mecC* (+), SCC*mec*-*orfX* (+)—methicillin-resistant coagulase-negative staphylococci, other than *S. aureus*	
MET-OXA	*mecA*/*mecC* (+) without other diagnostic elements—indicates other MRCoNS	
*Salmonella* sp.	*invA* (+)*—Salmonella enterica*	7041010 ^(9)^ 7041011 ^(10)^
*Campylobacter* sp.	16S rDNA sequence detected for *Campylobacter jejuni*	
*Yersinia* sp.	*ystB* (+)*—Yersinia enterocolitica*	
ESBL	[*bla*_CTX-M-A/B_, *bla*_SHV_ and *bla*_TEM_] (+)—β-lactamase activity for *Klebsiella pneumoniae* and *E. coli* about hospital origin *	7041069 ^(11)^
*Mcr*	*mcr*1 (+)*—*colistin resistant strains *E. coli*, *S. enterica*, *K. pneumoniae* and *Enterobacter* spp.	
*Legionella* sp.	*mip* conserved fragment detected—*Legionella pneumophila*	7041019 ^(12)^
MTBC	[IS*6110* and IS*1081*] (+)—*Mycobacterium tuberculosis* complex (*M. bovis*, *M. africanum*, *M. microti*, *M. caprae*, *M. pinnipedii*, *M. canetti* and *M. mungi*); other than MTB	7041071 ^(13)^ 7041048 ^(14)^
MTB	[IS*6110*, IS*1081*, TbD1 region of 16SrDNA sequence for *Mycobacterium* spp.] (+)—*Mycobacterium tuberculosis*	
NTM	[16S rDNA sequence for *Mycobacterium* spp.] (+)—*Mycobacteria* other than tuberculosis (MOTT)	

*—not excluded; the manufacturer of the tests does not exclude the presence of a specific serotype, strain, species or genus and suggests additional confirmation; the test may be a reference base for initial analysis for the additionally mentioned biological factors; **—Vitassay Healthcare, S.L.U, Huesca, Spain; Viasure V-smart (VS), multiplex qPCR tests, Certest Biotec S.L, San Mateo de Gállego, Spain. References to IVD tests: (1) vitassay.com/producto/vitassay-qpcr-ehec-epec-eiec-copia/; (2) vitassay.com/producto/vitassay-qpcr-e-coli-pathotypes-copia/; (3) vitassay.com/producto/vitassay-qpcr-vancomycin-resistanc-copia/; (4) www.certest.es/products/vancomycin-resistance-bd-maxtm-system/; (5) vitassay.com/producto/vitassay-qpcr-carbapenemase-generating-enterobacteriaceae-copia/; (6) www.certest.es/products/carbapenemase-producing-enterobacteriaceae/; (7) vitassay.com/producto/vitassay-qpcr-mrsa-copia/; (8) www.certest.es/products/methicillin-resistant-staphylococcus-aureus/; (9) vitassay.com/producto/vitassay-campylobacter-salmonella-shigella-eiec-copia/; (10) vitassay.com/producto/vitassay-qpcr-campylobacter-salmonella-yersinia-enterocolitica-copia/; (11) vitassay.com/producto/vitassay-qpcr-%ce%b2-lactamases-colistin-resistance-copia/; (12) vitassay.com/producto/vitassay-qpcr-l-pneumophila-copia/; (13) vitassay.com/producto/vitassay-qpcr-mtb-complex-copia/; (14) vitassay.com/producto/vitassay-qpcr-mtbc-ntm-copia/ (all accessed on 20 June 2026).

**Table 2:** Frequency of positive qPCR reactions for given resistance trait or presence of pathogens (*p* ≤ 0.0002; F ≤ 2.9608).

Parameter	Mean	Confid. (−/+) 95%		Sum	Variance	St. Dev.	St. Error
Raw wastewater of the municipal catchment (AH + HEs + HCUs + WTPs; N = 324)							
EHEC	0.7083	0.6008	0.8159	51	0.2095	0.4577	0.0539
STEC	0.2778	0.1718	0.3838	20	0.2034	0.4510	0.0532
EPEC	0.7917	0.6956	0.8878	57	0.1673	0.4090	0.0482
ETEC	0.7500	0.6475	0.8525	54	0.1901	0.4361	0.0514
*Shigella* EIEC	0.4583	0.3404	0.5762	33	0.2518	0.5018	0.0591
*Shigella* T1	0.3889	0.2735	0.5042	28	0.2410	0.4909	0.0579
*vanA*	0.8333	0.7451	0.9215	60	0.1408	0.3753	0.0442
***vanB***	1.0000	--	--	72	0.0000	0.0000	0.0000
NDM	0.7778	0.6794	0.8762	56	0.1753	0.4187	0.0493
**VIM**	1.0000	--	--	72	0.0000	0.0000	0.0000
OXA	0.9306	0.8704	0.9907	67	0.0655	0.2560	0.0302
**KPC**	1.0000	--	--	72	0.0000	0.0000	0.0000
IMP	0.9167	0.8513	0.9821	66	0.0775	0.2783	0.0328
*Campylobacter* sp.	0.9028	0.8327	0.9729	65	0.0890	0.2983	0.0352
*Salmonella* sp.	0.3889	0.2735	0.5042	28	0.2410	0.4909	0.0579
*Yersinia* sp.	0.7778	0.6794	0.8762	56	0.1753	0.4187	0.0493
MRSA	0.3056	0.1966	0.4146	22	0.2152	0.4639	0.0547
MSSA	0.3472	0.2346	0.4599	25	0.2299	0.4794	0.0565
MRCoNS	0.6944	0.5854	0.8035	50	0.2152	0.4639	0.0547
MET OXA	0.2639	0.1596	0.3682	19	0.1970	0.4438	0.0523
Treated wastewater discharged into river receivers (OFs; N = 72)							
EHEC	0.2115	0.0967	0.3263	11	0.1701	0.4124	0.0572
STEC	0.1731	0.0667	0.2794	9	0.1459	0.3820	0.0530
EPEC	0.4423	0.3027	0.5819	23	0.2515	0.5015	0.0695
ETEC	0.3654	0.2300	0.5008	19	0.2364	0.4862	0.0674
*Shigella* EIEC	0.8846	0.7948	0.9744	46	0.1041	0.3226	0.0447
*Shigella* T1	0.0577	0.0079	0.1232	3	0.0554	0.2354	0.0326
*vanA*	0.1346	0.0387	0.2306	7	0.1188	0.3446	0.0478
***vanB***	0.9423	0.8768	1.0079	49	0.0554	0.2354	0.0326
NDM	0.1731	0.0667	0.2794	9	0.1459	0.3820	0.0530
VIM	0.5385	0.3983	0.6786	28	0.2534	0.5034	0.0698
OXA	0.2692	0.1445	0.3939	14	0.2006	0.4479	0.0621
KPC	0.0769	0.0020	0.1518	4	0.0724	0.2691	0.0373
IMP	0.8846	0.7948	0.9744	46	0.1041	0.3226	0.0447
*Campylobacter* sp.	0.2308	0.1123	0.3492	12	0.1810	0.4254	0.0590
*Salmonella* sp.	0.0962	0.0133	0.1790	5	0.0886	0.2977	0.0413
*Yersinia* sp.	0.2885	0.1611	0.4158	15	0.2093	0.4575	0.0634
MRSA	0.0000	--	--	0	0.0000	0.0000	0.0000
MSSA	0.1731	0.0667	0.2794	9	0.1459	0.3820	0.0530
MRCoNS	0.2692	0.1445	0.3939	14	0.2006	0.4479	0.0621
MET OXA	0.1154	0.0256	0.2052	6	0.1041	0.3226	0.0447

**Bold**—the most common factor; underlined—the least common factor.

**Table 3:** Frequency of positive reaction for tubercle/non-tubercle bacillus analysis in samples on inlet CPs to wastewater treatment plants (N = 70).

Parameter/Cps	NTM	MTBC	MTB	Sum (% of TB */NTM)
WTP1	10	4	2	16 (37)
WTP1a	9	5	1	15 (40)
WTP2	11	3	2	16 (31)
WTP3	8	6	2	16 (50)
WTP4	12	2	0	14 (14)
Sum (%)	50 (71)	20 (28)	7 (10)	

* MTBC + MTB = TB. WTP1 and WTP1a—inflows of raw sewage to WWTP1; WTP2 and WTP3—inflows of raw sewage to wastewater treatment plants 2 and 3; WTP4—inflows of raw sewage to fourth wastewater treatment plant as the control; NTM—non-tuberculous mycobacteria; MTBC—*Mycobacterium tuberculosis* complex; MTB—*Mycobacterium tuberculosis*; TB—tubercle bacillus.

**Table 4:** Frequency of main species as number of reads after 16S rDNA sequencing for the most abundant strains in samples from Warsaw’s collection points during peak periods of two COVID-19 waves, 2023 and 2024 (N = 70).

Species	AH	HCU	HE	WTP	OF	Mean	Confid (−95%)	Confid (+95%)	Median	St.Dev.
*Aliarcobacter skirrowii*	73,360 *	216,182 *	108,025 *	705,624 *	31,383 *	226,914.8	−116,077	569,906.5	108,025 **	276,235.5
*Arcobacter porcinus*	15,886	52,154	50,837	283,090	12,977	82,988.8	−57,803.9	223,781.5	50,837	113,390.3
*Agathobacter rectalis*	24,945 **	212,549	22,933	82,774	4723	69,584.8	−36,118.8	175,288.4	24,945	85,130.59
*Faecalibacterium prausnitzii*	12,581	174,697	33,881	66,210	1859	57,845.6	−28,808.6	144,499.8	33,881 **	69,788.79
*Faecalibacterium duncaniae*	12,852	169,555	28,744	59,801	1358	54,462	−29,952.3	138,876.3	28,744	67,984.83
*Faecalibacterium longum*	15,170	158,457	21,285	43,131	532	47,715	−31,471.5	126,901.5	21,285	63,774.53
*Aliarcobacter cryaerophilus*	7544	29,283	30,302	160,218	8227	47,114.8	−32,562.6	126,792.2	29,283	64,169.87
*Segatella copri*	4337	55,753	68,038	80,208	1836	42,034.4	−3412.1	87,480.9	55,753 **	36,601.29
*Arcobacter suis*	6059	12,266	7258	128,045	7317	32,189	−34,411.7	98,789.68	7317	53,638.25
*Pseudaeromonas sharmana*	4823	34,368	36,021	71,536	3500	30,049.6	−4609.79	64,708.99	34,368 **	27,913.66
*Faecalibacterium butyricigenerans*	6844	86,158	15,351	32,899	389	28,328.2	−14,575.5	71,231.94	15,351	34,553.42
*Aeromonas eucrenophila*	7967	67,688	16,161	43,008	3833	27,731.4	−5848.34	61,311.14	16,161 **	27,044.15
Mean	16,030.67	105,759.2	36,569.67	146,378.7 ***	6494.5	* the most abundant in the sample; ** basis to select strains for further characterization; *** SCP with the highest probability of presence all selected predominants AH (transportation hub); HCU (healthcare units); HE (mixed catchment area incl. housing estates and military units); WTP (inflow to wastewater treatment plants); OF (outflow from WWTPs)				
Confid (−95%)	3956.952	58,023.46	18,852.46	26,108.52	986.2749					
Confid (+95%)	28,104.38	153,494.9	54,286.87	266,648.8	12,002.73					
Median	10,274	76,923	29,523	75,872	3666.5					
St.dev.	19,002.66	75,130.61	27,884.88	189,291.6	8669.323					

## Data Availability

The data presented in this study are openly available in the European Nucleotide Archive under BioProject PRJEB95765. Additional datasets generated and/or analyzed in this study are available from the corresponding author on reasonable request.

## Abbreviations

The following additional abbreviations are used in this manuscript:

IBD-EIM	Inflammatory bowel disease associated with extra-intestinal manifestations
ASV	Amplicon sequence variants
UC-EIM	Ulcerative colitis with extra-intestinal manifestations

## Appendix A

**Table A1:** Collection points with technical characterization and sampling methodology.

Names of Representative Collection Points (CPs)		Technical Characteristics of Catchment and CPs with Population Equivalent (p.e.)	Sampling Method
AH Transportation hub (raw sewage)		Shared transport and handling facilities, including the infrastructure of two international airports, railways and sub-stations handling passenger traffic, with communication between airports	collected manually between 7:00 AM and 4:00 PM from inspection wells once in a given single collection point per working day (qualified single samples)
HCU Healthcare units (raw sewage)	1	The medical complex of 13 departments (>700 beds) and the communicable diseases division	
	2	The medical Research Institute of the military with 6 centers and 25 clinics	
	3	The medical Research Institute with 23 clinics, including neurological and psychiatric divisions	
	4	Communicable diseases center with 11 departments, labs and wastewater pretreatment	
HE Housing estates with military area (raw sewage)	1	Ten sub-HEs and selected airport facilities—130,000 p.e.	
	2	Eleven sub-HEs with pharmacy and cement industry—159,000 p.e.	
	3	Part of the northeast housing estate with 30% of green complex—60,000 p.e.	
	4	Part of the northeast housing estate with industries and stores—60,000 p.e.	
	5	Fourteen sub-HEs with fully complex municipal infrastructure—150,000 p.e.	
	6	Eleven sub-HEs in the southeast of Warsaw with 7 railway stations—45,000 p.e.	
	7	Eleven sub-HEs in the southeast of Warsaw with flood embankment facilities—43,000 p.e.	
	8	Nine sub-HEs with military facilities, research institutes, and an education hub—51,000 p.e.	
WTP Inflows to wastewater treatment plants * from the given catchment area (raw sewage)	1	The left bank of the Vistula River and the north of Warsaw (8 districts)—inflow ca.180,000 m^3^/d	FPM (150 mL/30 min)
	1a	The right bank of the Vistula River (8 districts)—inflow ca. 240,000 m^3^/d	
	2	The left-south bank of the Vistula River—ca. 60,000 m^3^/d	every hour per day
	3	The northwest of Warsaw at the left bank of the Vistula River—ca. 40,000 m^3^/d	FPM (150 mL/30 min)
	4	The north districts of Warsaw, out of the Capital City—background inflow ca. 5300 m^3^/d	FPM (100 mL/30 min)
OF Outflows from wastewater treatment plants (treated sewage)	1	Directly to the Vistula River from WWTP1 (left and right inflows)	TPM (150 mL/500 m^3^)
	2	Directly to the Vistula River from WWTP2	FPM (120 mL/1000 m^3^)
	3	Directly to the Utrata River from WWTP3	FPM (100 mL/100 m^3^)
	4	Directly to the Narew River from WWTP4 (background outflow)	

* Wastewater Treatment Plant (WWTP) characteristics (based on Municipal Water and Sewerage Company of Warsaw (MWSCW). “Czajka” plant. MPWiK 2025. https://www.mpwik.com.pl/view/zaklad-czajka, accessed on 9 May 2026): WWPT1 uses mechanical and biological processes, divided into 10 technological lines including a biological reactor with activated sludge of the BIODENIPHO type, two secondary clarifiers, and biogas production. Treated wastes were discharged directly into the Vistula River; WWTP2 operates as a fully hermetic facility based on mechanical facilities and bioreactors with oxygen–anoxic zones for the biological removal of nitrogen and phosphorus using activated sludge microorganisms; WWTP3 technologically included bioreactors with oxygen–anoxic zones for the biological removal of nitrogen and phosphorus using activated sludge microorganisms supported by secondary clarifiers; WWTP4, “external background”, was based on activated sludge technology, adapted to mechanical–biological–chemical treatment. FPM—flow-proportional mode, TPM—time-proportional mode.

**Table A2:** Organization of samples for bacterial 16S rDNA sequencing in sewage.

Names of Collection Points (CPs)	Auxiliary Markings for Sequencing Purposes	TNA Quality [A260/280]	TNA Quantity [ng/µL]	Pools Representing Mixed COVID-19 Waves	COVID-19 Wave
AH	AH_1	2.3	156		“2023 wave” of COVID-19 in range of maximum SARS-CoV-2 cases 4 December 2023; 11 December 2023; 9 January 2024
HCU_1	HCU_1	2.3	127		
HCU_2	HCU_2	2.3	71		
HCU_3	HCU_3	2.3	87	HCU_3	
HCU_4	HCU_4	2.3	259		
HE1–8	HE_1	2.3	337		
WTP_1-1a	WTP_1	2.3	66		
WTP_2	WTP_2	2.3	62		
WTP_3	WTP_3	2.3	60		
WTP_4	WTP_4	2.3	57	WTP_4	
OF_1	OF_1	2.0	19		
OF_2	OF_2	2.0	18		
OF_3	OF_3	1.8	74	OF_3	
OF_4	OF_4	1.9	31	OF_4	
AH	AH_2	2.2	60		“2024 wave” of COVID-19 in range of maximum SARS-CoV-2 cases 6 August 2023; 13 August 2024; 3 September 2024; 17 September 2024; 24 September 2024
HCU_1	HCU_5	2.3	117		
HCU_2	HCU_6	2.3	64		
HCU_3	HCU_7	2.3	75	HCU_7	
HCU_4	HCU_8	2.3	81		
HE1-8	HE_2	2.3	206		
WTP_1-1a	WTP_5	2.4	32		
WTP_2	WTP_6	2.4	38		
WTP_3	WTP_7	2.4	26		
WTP_4	WTP_8	2.3	53	WTP_8	
OF_1	OF_5	2.2	18		
OF_2	OF_6	2.1	12		
OF_3	OF_7	2.1	22	OF_7	
OF_4	OF_8	2.2	18	OF_8	

**Table A3:** Per-sample read counts.

Collection Points (Barcodes)	Reads (800–2000 bp)	Unclassified	Classified (Total Counts)	Mean Length [bp]	Median Length [bp]	Min [bp]	Max [bp]	Mean Q-Score	Median Q-Score
AH1	391	0	178	1492.5	1587	828	1851	12.8	12
AH2	619,218	1	609,294	1579.6	1593	800	2000	14.5	14
HCU1	539,296	0	528,663	1587.9	1593	800	2000	14.5	14
HCU2	671,645	0	662,821	1578.5	1591			14.4	
HCU3	552,669	1	545,851	1581.2	1593			14.5	
HCU4	557,139	0	549,823	1583.5	1592			14.5	
HCU5	453,965	1	445,717	1580.9	1593			14.5	
HCU6	484,548	0	471,202	1582.7	1593			14.5	
HCU7	583,642	1	575,736	1585.9	1597			14.5	
HCU8	279,672	0	274,543	1579.9	1589			14.6	
HE1	798,820	0	789,877	1583.4	1595	800	2000	14.5	14
HE2	693,424	2	684,596	1579.2	1589		1999	14.5	
WTP1	519,562	0	511,006	1578.8	1590	800	2000	14.5	14
WTP2	488,924	0	480,485	1578.3	1589		2000	14.5	
WTP3	258,632	0	250,409	1576.8	1590		1998	14.6	
WTP4	554,564	0	543,983	1581	1591		1995	14.6	
WTP5	595,402	0	581,658	1582.2	1593		2000	14.6	
WTP6	743,346	1	732,100	1582.6	1592		1998	14.5	
WTP7	40,636	1	36,199	1583.3	1599		2000	14.5	
WTP8	50,629	10	42,760	1585.9	1596		1993	14.5	
OF1	116,103	18	103,630	1584.5	1595	800	1998	14.5	14
OF2	88,736	11	79,036	1585.1	1596	800	1996	14.5	
OF3	181,850	9	169,531	1585.3	1595	800	1999	14.5	
OF4	26,950	8	19,634	1596.3	1599	801	1999	14.5	
Sum	9,899,763	64	9,688,732						
